# Editorial: The changing focus of regulatory frameworks around the globe and the opportunities for harmonization

**DOI:** 10.3389/fmed.2025.1645278

**Published:** 2025-07-10

**Authors:** Armando Magrelli, Daniel J. O'Connor, Violeta Stoyanova-Beninska

**Affiliations:** ^1^National Center for Drug Research and Evaluation, Istituto Superiore di Sanità, Rome, Italy; ^2^The Association of the British Pharmaceutical Industry (ABPI), London, United Kingdom; ^3^European Medicines Agency, Amsterdam, Netherlands

**Keywords:** regulatory science, medicines regulatory agencies, regulatory convergence, orphan medicinal product (OMP), the international conference of harmonization (ICH), ICMRA, regulatory sandboxes, innovative methodologies

## Regulatory framework

The regulatory environment is key for protecting public health and has become more complex and sophisticated in recent years, paralleling the advances in scientific discovery and 21^st^ century technological opportunities. The pharmaceutical field and medicines and medical devices development is increasingly global, and multinational firms must make informed and often challenging decisions about where and when to locate their activity. It is vital that regulators provide the most effective approval systems that are proportionate and committed to patient safety and timely access, whilst supporting innovative research and efficient development programmes.

Increasingly, alongside bespoke regulatory initiatives found across different jurisdictions that reflect particular population or public health needs and life sciences ambitions, *reliance* (whereby the national regulatory authority in one jurisdiction may take into account and give significant weight to assessments performed by another national regulatory authority or trusted institution, or to any other authoritative information in reaching its own decision), *harmonization* (process by which technical guidelines are developed to be uniform across participating authorities) and *convergence* (process whereby the regulatory requirements across countries or regions become more similar or “aligned” over time) are seen as growing themes. A number of “tools” exist to facilitate these more aligned approaches, including work at the International Council for Harmonization of Technical Requirements for Pharmaceuticals for Human Use (ICH) and the International Coalition of Medicines Regulatory Authorities (ICMRA). It is also increasingly recognized that Regulatory Science plays an important role in adapting the global regulatory frameworks which exist to ensure that patients can access high quality medical technologies and public confidence in regulatory frameworks is maintained and improved.

## Regulatory science and novel regulatory concepts

Regulatory Science can be defined as a range of scientific disciplines that are applied to the quality, safety and efficacy assessment of medicinal products and that inform regulatory decision-making throughout the lifecycle of a medicine, encompassing basic and applied medicinal science and social sciences, which contribute to the development of regulatory standards and tools. Regulatory Science is expected to be responsive to emerging changes in technology, clinical practice and societal and public health needs. Progress has been made in the application and evolution of regulatory (legislative) procedures for the benefit of patients and public health but also in driving and enabling innovation.

The current global landscape of medicines is changing rapidly with the increasing focus on gene editing techniques, specific modifications allowing for individualized medicines or targeted therapies for very small number of individuals. The conventional regulatory paths are mostly positioned for larger populations and may not be suitable for these type of approaches.

Regulatory sandboxes have emerged as an innovative mechanism to facilitate the development and approval of new technologies, including pharmaceuticals. A regulatory sandbox is an environment where firms can test new innovations under the supervision of a regulator. The aim is simple: to facilitate innovation in a safe and responsible manner. Innovations that can be tested include new products, services, solutions, technologies, business models and even policies. The application of regulatory sandboxes in the context of rare disease therapies presents a promising avenue to accelerate the development, approval, and access to disease-modifying and life-saving therapies. Given the complexities of rare diseases and the regulatory hurdles faced by orphan medicinal products, regulatory sandboxes offer a structured yet flexible environment where new regulatory approaches can be tested and refined. The concept of regulatory sandboxes however is not limited to regulation of medicines for rare diseases and is being explored in a broader context in any situations where the established regulatory pathways might not be fit for purpose.

## Research Topic—evolving regulatory processes

Our Research Topic covered elements of the' Changing Focus of Regulatory Frameworks Around the Globe and the Opportunities for Harmonization'. The Research Topic includes 10 papers from across the globe including Europe, Africa and Japan, exemplifying the diversity of the changing practice and emphasis of regulatory science, with the need to foster more harmonized and convergent approaches across the globe.

Scientific advice from competent authorities is a critical tool that helps innovators navigate the complexities of the regulatory requirements in medicines development. Gravanis et al. discuss the challenges and ongoing initiatives toward better integrated EU scientific advice, noting the increasing importance of parallel advice with other decision makers such as Health Technology Assessment (HTA bodies) and the need to forge closer links with medical device regulators. Despite the benefits of scientific advice to developer, patient, payer and regulator, there are challenges, and these include aspects such as the need to carefully manage the separation between individuals in prominent roles during early advice and later assessment, whilst being cognisant of capacity concerns and existing resource constraints in the EU medicines regulatory network.

Another important regulatory tool for innovators is qualification procedures of novel methodologies such as non-clinical and *in vitro* models, biomarkers and pharmacometric methods. Giannuzzi et al. analyse EMA qualification procedures and explore innovative research methodologies in the EU regulatory framework from a pediatric perspective. They found that only 6 out of 27 qualification procedures reported pediatric data, despite the fact that many more of these 27 procedures hold significant promise for application in the pediatric population. This study reiterates the call to strengthen the framework for pediatrics, which despite specific regulatory provisions, is often still a neglected area of research and development.

Capacity issues and immature regulatory systems can be detrimental for provision of timely access to medicines and other health technologies. Regulatory reliance provides one solution, described as the act whereby the regulatory authority in one jurisdiction takes into account and gives significant weight to assessments performed by another regulatory authority or trusted institution, or to any other authoritative information in reaching its own decision. Broojerdi et al. describe evidence-based approaches for promoting regulatory reliance, allowing for increased access to quality-assured medical products. A number of key recommendations are put forward to further improve and build the sustainability of the WHO-listed authorities framework, supporting the advancement of regulatory outputs and outcomes, and generating a positive impact on global public health.

Wens et al. tackle the challenging topic of defining unmet medical needs from a regulatory perspective, an increasingly important area of focus for the new EU pharmaceutical legislation and the linkage to bespoke pathways and incentives. In responses to a survey of stakeholders, areas of agreement and disagreement are elucidated with a clear recommendation for the need for further discussion on the proposed criteria for unmet medical need in order to avoid ambiguity and maximize the potential opportunities for patients.

Medicines regulatory harmonization provides several benefits including improving public health through faster availability of safe, high-quality, and effective medical products. It also enhances the standardization of technical guidelines and facilitates work-sharing among regulatory authorities. Ngum et al. compared the review models, target timelines and data requirements used in assessing applications by the East African Community Medicines Registration Harmonization (EAC-MRH) initiative. Their study led to several recommendations aimed at improving current registration processes, minimizing the duplication of limited resources, and reducing costs and burdens for the pharmaceutical industry.

In this Research Topic, Brown et al. highlight key advancements and ongoing challenges in regulatory and market access for rare disease medicines. Legislative initiatives such as the U.S. Orphan Drug Act and the EU Orphan Drug Regulation have significantly increased approvals of orphan drugs. Despite these regulatory successes, global patient access remains uneven due to fragmented pricing and reimbursement systems. Brown et al. emphasizes the need for collaborative efforts to bridge these gaps and translate innovation into tangible relevant outcomes for patients.

Similarly, Owusu-Asante et al. examine the status and improvement opportunities for Good Review Practices (GRevPs) in seven West African countries under the ECOWAS Medicines Regulatory Harmonization initiative. Their analysis identifies disparities in regulatory autonomy, transparency, and communication, highlighting Sierra Leone's notable dedication to continuous enhancement of regulatory review processes aligned with GRevP principles.

Building on these insights, Hassen et al. stress that ensuring pharmaceutical quality remains a significant public health issue in Africa, exacerbated by weak regulatory frameworks, limited resources, and corruption. They advocate adopting Quality by Design, embedding quality assurance throughout pharmaceutical production, to improve product reliability and safety. The study underscores the urgent need for African nations to harmonize regulations, enhance enforcement, and invest in regulatory infrastructure to protect public health.

Post marketing risk management measures hold an important place in managing uncertainties about the safety profile of medicines at the time of their marketing authorization. Different jurisdictions have introduced rules and actions with the scope to minimize risks, but also to follow up, identify and assess safety signals. Kameyama et al., have provided a review of the current situation and issues regarding termination of risk management plans in Japan. A retrospective analysis of a 10 year period (2013–2023) has shown that out of 72 drugs with RMPs completed re-examination, the RMP requirement was lifted for 69 drugs (95.8%) and remained for three drugs (4.2%) only. Since after removal of the RMP requirement there is limited information regarding risks that take time to manifest or insufficient information regarding safety during long-term administration, the authors call for reconsideration of the application of this rule. In addition, the authors emphasize the differences with EU and US legislations regarding pharmacovigilance and risk minimization activities, where such termination of RMP is not in place.

In conclusions the authors point to the potential source of confusion because medicines marketed in several parts of the world must comply with divergent regulatory requirements. With an increasing trend for globalization, aligning of regulatory RMP requirements across jurisdictions worldwide could help in global availability of medicines and it will guarantee better drug safety management.

Samukange et al. use the World Health Organization's Global Benchmarking Tool Plus Blood (GBT + Blood)—a recognized framework that provides detailed sub-indicators for evaluating specific regulatory functions related to blood, blood components, and plasma-derived products. This tool is widely used to assess the maturity of national regulatory systems and guide improvements in regulatory oversight. The authors have compared WHO-designated maturity level 3 (ML3) competent national regulatory authorities (NRAs) with non-designated NRAs. The results clearly indicate a disparity between the registration/marketing authorization function and the approval process for blood products. The authors concluded that there is an urgent need to prioritize and strengthen regulatory capacities, particularly in the approval process for blood products.

## Summary and future perspectives

Medicines and medical technology development is a global endeavor and exchange of experience and knowledge between regulatory agencies working in different jurisdictions is not only necessary but seen increasingly as essential.

The publications included in this Research Topic provide an opportunity for focused discussion on specific aspects of the regulatory practices in certain parts of the world or the use of certain regulatory tools. While each individual publication tackles a specific aspect either in regulatory support in drug development, or in approval and post marketing safety monitoring, the common theme emerging are the gaps and divergences identified, and the proposal for re-thinking of the system to drive efficiencies, harmonization and reduce duplication. Although not comprehensive, this collection provides readers with a curated set of challenges and opportunities, highlighting the need for continued reflection and the exploration of alternative and innovative approaches in regulatory science and practice. The guest editors truly hope that this has opened the door for debate and future research in the domain of regulatory science, and it's positive growing impact on public health.

